# A dataset for assessing phytolith data for implementation of the FAIR data principles

**DOI:** 10.1038/s41597-023-02296-8

**Published:** 2023-07-21

**Authors:** Céline Kerfant, Javier Ruiz-Pérez, Juan José García-Granero, Carla Lancelotti, Marco Madella, Emma Karoune

**Affiliations:** 1 Universitat Pompeu Fabra, Department of Humanities, Barcelona, 08005 Spain; 2 Texas A&M University, Department of Ecology and Conservation Biology, College Station, TX 77843 Texas USA; 3 HUMANE—Human Ecology and Archaeology, IMF, CSIC, 08001 Barcelona, Spain; 4 ICREA, Catalan Institution for Research and Advanced Studies, Barcelona, 08010 Spain; 5 Historic England, Investigative Science Team, Portsmouth, PO4 9LD England

**Keywords:** Research management, Plant sciences

## Abstract

Phytolith research contributes to our understanding of plant-related studies such as plant use in archaeological contexts and past landscapes in palaeoecology. This multi-disciplinarity combined with the specificities of phytoliths themselves (multiplicity, redundancy, naming issues) produces a wide variety of methodologies. Combined with a lack of data sharing and transparency in published studies, it means data are hard to find and understand, and therefore difficult to reuse. This situation is challenging for phytolith researchers to collaborate from the same and different disciplines for improving methodologies and conducting meta-analyses. Implementing The FAIR Data principles (Findable, Accessible, Interoperable and Reusable) would improve transparency and accessibility for greater research data sustainability and reuse. This paper sets out the method used to conduct a FAIR assessment of existing phytolith data. We sampled and assessed 100 articles of phytolith research (2016–2020) in terms of the FAIR principles. The end goal of this project is to use the findings from this dataset to propose FAIR guidance for more sustainable publishing of data and research in phytolith studies.

## Background and Summary

**Fig. 1:**
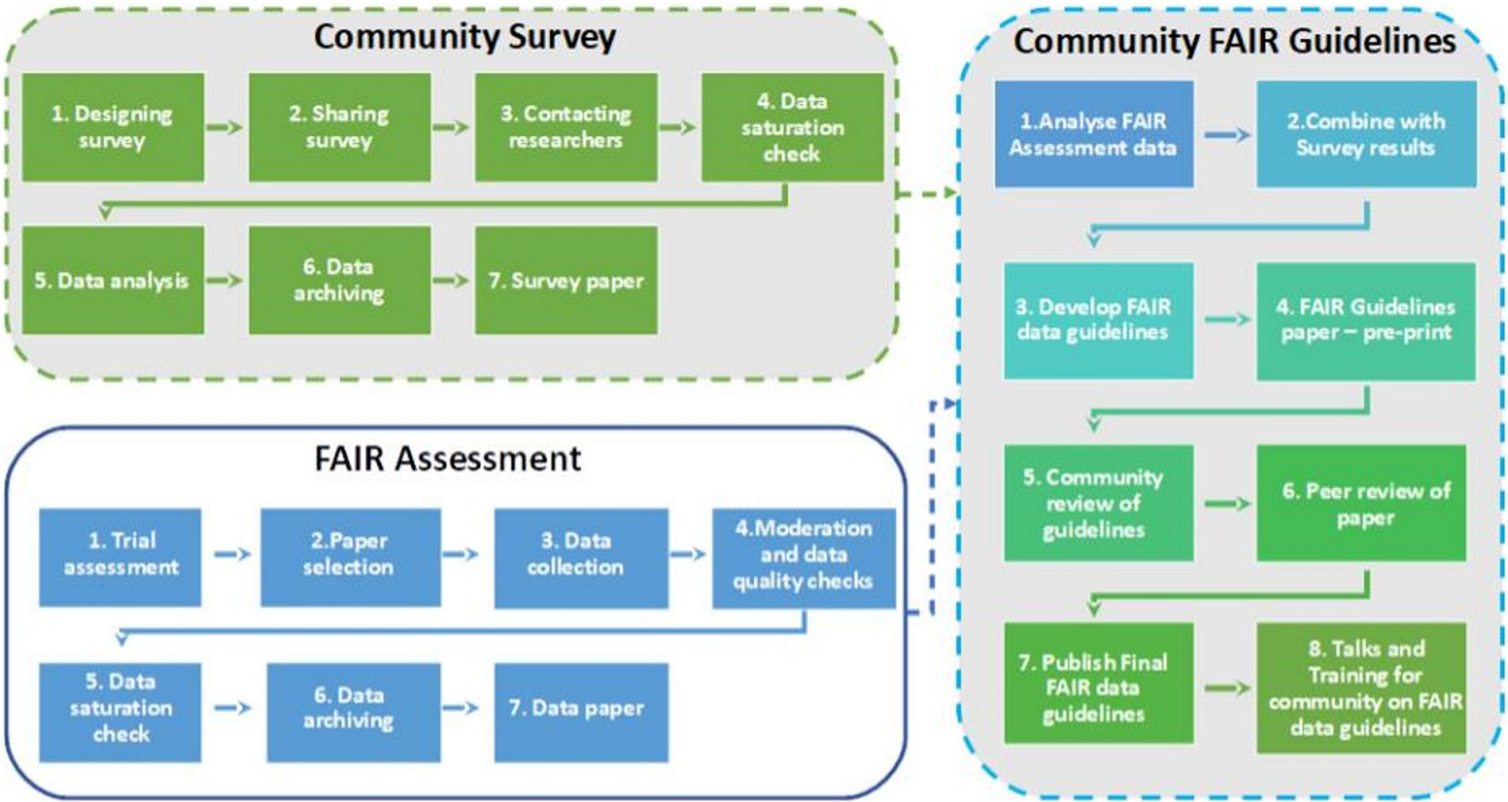
FAIR Phytoliths project workflow - the FAIR assessment is the workflow for the data paper and will be combined with the survey paper results to produce the first draft of the Community guidelines. Dotted lines indicate work packages still to be completed on the FAIR Phytoliths Project.

FAIR stands for Findable, Accessible, Interoperable and Reusable. The FAIR guiding principles^1^ were developed to maximise the sustainability and reuse of data as a framework for data management and stewardship. FAIR aims to make data available whenever possible, and when it is not, metadata is required to allow a full understanding of the data. For example, if data cannot be openly accessible, metadata describes the data and therefore enables the findability, accessibility, interoperability and reusability of these datasets^1,2^. Metadata plays an important role in each step of the FAIRification process, including both human- and machine-readable metadata^3,4^. The FAIR principles can be applied to create FAIR standardised tools for self and community research assessment^1,2,5^, which can be used to improve (meta)data quality and searchability^5^, as well as reproducibility^6^ of research (Fig. 1).

This dataset was developed as part of the ‘Increasing the FAIRness of phytolith data’ Project (commonly known as the FAIR Phytoliths Project)^7^, which is a community initiative to address the lack of data sharing and the difficulty of reusing data in phytolith research. Phytoliths are microscopic bodies of silica that develop within living plants from the uptake of groundwater^8–11^. In the last two decades, phytolith research has evolved quickly and is used in a wide range of scientific disciplines including archaeobotany, palaeobotany, geology and plant physiology, among others^12–19^.

However, this increased application of phytolith analysis in a variety of disciplines has resulted in a dispersion of terminologies and methods^10,20–23^. Two nomenclatures (ICPN 1.0 and 2.0^8,10^) exist to standardise phytolith naming but these are limited to certain morphotypes and taxonomic groups. The wide range of phytolith methodologies and applications, as well as the diverse background of phytolith researchers, makes collaborative work difficult and hampers the sharing and reusability of data. In fact, a recent study looking at open science practices in phytolith research revealed a clear lack of data sharing (53% shared data in any format) and data reusability (only 4%) from a sample of 341 published papers with primary phytolith data^23,24^.

The FAIR Phytoliths Project focuses on examining published phytolith research in terms of how data sharing and management can be improved using the FAIR principles as a framework. Inspired by the wheel of FAIRification^25^ and the need to define objects and variables to be fairified in the pre-FAIRifying stage, we have gathered a wide sample of phytolith research from different disciplines to assess the full extent of published phytolith data. We chose to take published research articles from studies conducted in two geographic areas, Europe and South America, and published between 2016 and 2020 (both included). These two regions were chosen because they have different traditions of phytolith methodologies (e.g., counting strategies, nomenclatures) and therefore a selection of papers from both regions was thought to give suitable breadth of published phytolith data.

Once we had selected the published articles, we scrutinised all aspects of the published material to highlight what elements were already in line with the FAIR principles and how other elements could be improved. The results of this dataset will be used to develop domain-specific FAIR guidelines for the phytolith community (with community review) and make a FAIR assessment tool to help phytolith researchers assess their own datasets and publications. Following this study, we will develop training materials for phytolith researchers so that a wide range of tools will be available to improve data sharing and collaboration among phytolith researchers.

## Methods

The method for data collection used in the FAIR Phytoliths Project is a development of the method created by Karoune^23,24^ to assess open science practices within phytolith research. It is designed to provide a transparent record of the FAIR assessment dataset and aims to be reproducible. The full data set is available here 10.5281/zenodo.7851930^26^.

Six phytolith researchers (Emma Karoune, Carla Lancelotti, Juan José García-Granero, Javier Ruiz-Pérez, Marco Madella, Céline Kerfant) conducted the whole study in two steps: A first team of five assessors carried out the trial of data collection methodology and a second team of four assessors conducted the main data collection. One of them did not take part in the trial assessment.

We have developed a transparent methodology in this study to ensure trust and a deeper understanding of this project’s work within our community. This will hopefully generate greater uptake of the resulting guidelines and therefore initiate a movement to the desired improvement in data management and stewardship within the phytolith community.

The full description of the experimental design is grouped into two sections: trial of data collection methodology and data collection.

### Trial of data collection methodology

An initial method for data collection was developed based on previous work by Karoune^23,24^. A data categories table and a matching Google Form^26^ were created. The terms *data categories* and *variables* are being used interchangeably in this article - the data dictionary has been standardised using the term variable but prior to this both terms will be used in this article.

The trial of data collection assessed the clarity of meaning for each variable before starting the FAIR assessment. The document containing the full details of the trial can be accessed through our repository^26^. The methodology for the data collection was evaluated, firstly, to check if any data categories (variables) were missing; secondly, to make sure that we were capturing all of the information needed for assessing all elements of the FAIR data principles in the selected articles; and, lastly, to determine if any of the categories produced a wide range of results among the assessors.

During the trial process we linked the FAIR principles to the variables that are specific for phytolith research. In the trial, the variables were ordered in terms of the FAIR acronym^26^.

The trial was conducted by five researchers. We each selected one article (five articles in total) from different world regions and different types of studies. We each assessed all five papers and used the Google Form to collect data. We then compared our results and discussed differences recorded for each article and any problems that we had with collecting data for each data category.

### Data collection methodology

The first step was to conduct two literature searches to create a sample of publications from Europe and South America. We standardised how we determined what countries are in Europe and South America according to Wikipedia:

- List of European countries: (https://en.wikipedia.org/wiki/List_of_European_countries_by_area)
- List of South American countries: (https://en.wikipedia.org/wiki/List_of_South_American_countries_by_area)

Due to multiple members of our team collecting data, we needed to get a static list of papers rather than using online searches. We used Publish or Perish software (version 0.7.2) (https://harzing.com/resources/publish-or-perish) to run two keyword searches (‘phytoliths’ and ‘Europe’ or ‘South America’ with a date range of 2016 to 2020, both included) using the Google Scholar as search engine and then we exported the results as a .csv file. This produced a list of 989 articles for Europe and 996 for South America.

We selected the first 50 articles from both searches by manually looking at the articles to check they met our search criteria of being a phytolith article, in one of the chosen areas (Europe or South America) and a study with primary data on material. In the case of articles with two different publication dates (online publication date and final publication date), the online publication date was chosen.

Each article was then assessed using the prepared Google Form. We collected data in two spreadsheets - one for European publications and one for South America publications. Notes were put in a README file^26^ (there is one README file for each spreadsheet with the date, data collectors name and what was done in each working session). This made it easier for us to work asynchronously.

Once all the articles were assessed the R package *roadoi* version 0.7.2 (uses Unpaywall with R) was used on our list of articles to find information about the open access status of the articles. The documentation for the data categories in this package can be found here: (https://unpaywall.org/data-format). This information was integrated into the data sets after the rest of the data was collected.

Finally, the datasets were merged to create the final dataset for assessing FAIR practices in existing phytolith publications.

Standardisation and transparency of data collection was vital in this study, as the data collection was being conducted by four assessors working in a distributed team. Specifically, we needed to avoid duplication and control subjectivity in the data collection. This was achieved by using README files for each dataset during the data collection, so that each data collector recorded their work during every session. The progress, issues and changes of the FAIR assessment datasets were recorded in the README files for each dataset and were discussed every two weeks during online meetings as well as in a dedicated Slack channel.

We also collected data using Google Forms whereby most of the variables displayed a drop down list of responses that had to be selected^26^ and very few of the variables had open text boxes. This meant that there was little room for typing errors in the dataset and therefore less need for data cleaning before analysis. This also ensured that subjectivity in the answer was kept at a minimum and avoided the creation of many single-entry variables.

## Data Records

The entire dataset is deposited in the following Zenodo repository^26^, the structure of the data sheets is as follows.

Raw data files can be found in the project’s Zenodo repository^26^.This folder contains the two raw datasets for Europe and South America as .csv files and also the final combined dataset as a .csv file.

The Zenodo repository also contains a fully transparent record of data collection including methods development: data collection, data validation and data saturation checks^26^.

During data collection we used a working data dictionary to help us with standardisation of our decision in line with guidance from the FAIR Cookbook (https://fairplus.github.io/the-fair-cookbook).This has been changed into a standardised data dictionary^26^.

The first section of variables (1–7) gathers general information about each article such as journal name and full reference of the article. This section also includes the information about our sampling criteria, which comprises year of first publication online (between 2016 and 2020) and the location of the study in the article (either from an European or South American country, or countries, or regions using Geonames standardised list of countries or regions in each region). We also gathered information on time period (e.g., Holocene archaeological site, modern plant cultivation experiment) and type of study (e.g., archaeology, plant physiology). This was used at the analysis stage to group articles in different ways to look for any differences in alignment with the FAIR principles.

The second section (variables 8–11) captured information about open access. The use of open access publishing and type of journal in terms of openness may influence the way data is published as there are differences in publisher data policies such as in the policy of the journal Vegetation History and Archaeobotany authors are encouraged to deposit data that supports their findings, where as in Plos One authors are required to make all data necessary to replicate study’s findings publicly available without restriction^23^.

The third section focused on the methods reported in the articles (variables 12–16). For data to be reusable, full and transparent reporting of methods is required. For phytolith research, the method of extraction needs to be reported fully and this information is captured in variable 12. The “counting method” (variable 13) assesses the level of replicability of this analytical step as this is a paramount detail for comparing datasets or considering merging datasets. We looked for information included in the articles about the number of phytoliths counted per slide, if those phytoliths were single-celled or multi-celled, if unidentified phytoliths were represented in the count and if any morphometric study was performed. If any of this information was not included or unclear, then we did not consider it as a replicable counting method.

We also collected information about the instruments used (variable 14). We looked specifically for two pieces of information: the general type of microscope (not the brand or model of microscope) and the magnification used to carry out the counting.

We collected data on the use of specific nomenclatures (variable 15), such as ICPN 1.0^8^ and ICPN 2.0^10^, but also other naming conventions developed and used locally by specialists within their own laboratories^27–30^ or by researchers creating ad-hoc reference collections^31,32^. Specifically, variable 16 was used to collect data on whether the standard nomenclatures were being fully used - this meant if the articles showed no adaptations to the standard nomenclatures or these adaptations had been fully described. This was only done for those articles that had stated the use of either ICPN 1.0^8^ or ICPN 2.0^10^.

The last section (variables 17–25) aimed at collecting information on the data presented in or linked to the articles. We recorded where the data was located (variable 17) and, if in a repository, which repository (variable 18) was used, including institutional repository or long-term repository, among other possibilities.

The “type of data” (variable 19) and “data format” (variable 20) variables assess the data made available in or linked to the article for reuse. The only reusable data are raw data: in the case of phytolith research an editable file providing the sample weights (processing weights recorded during extraction of phytoliths from sediments or other materials) and the absolute counts of each morphotype in each of the samples. It is difficult to reuse processed and reorganised data as this limits the reuse potential of the data^23^. In this dataset, we therefore recorded the most reusable data presented in the article. This means there could have been different types of data in tables or graphs but we only recorded the most reusable data presented. When only graphs were presented in the article, we collected this as “no data provided-only graph” due to the difficulty in extracting data from graphs.

The data availability statement (variable 21) allows other researchers to easily find data presented in articles. We checked if a data availability statement was provided and, if so, made sure that the statement did relate to the data. Some data can be available but under some specific conditions and each of them have to be separately assessed. For example, some datasets can be available on request (from authors, organisation or third party), a practice that hampers data reusability as data and metadata are not easily accessible^33^. In other cases, data can be fully available but in different ways. They could be presented within the article, in a supplementary file or could be linked to the article with a DOI. All these possibilities were recorded.

The data licence (variable 22) needed to be assessed as it is the legal framework in which researchers will find indications about how to reuse the dataset and how to cite the dataset. Therefore, we recorded what kind of licence was specifically added to the data.

The presence or absence of phytolith microphotographs (variable 23) was also assessed. Illustrations are part of both ICPNs^8,10^ principles to describe phytolith morphotypes. This documentation aspect is important for confirming identifications of phytolith morphotypes and discussions of nomenclature standardisation. We recorded under three responses: none, only significant ones, and all. ‘Only significant ones’ were recorded when there were some photographs but not all the morphotypes found in the data.

The last data variable concerned the software used to carry out the statistical analysis (variable 24). We recorded any mentioned software used for statistical analysis in the paper. This is important to understand the types of analysis tools that are currently used on phytolith data and particularly the use of open source software that can aid reproducibility of research and reuse of data.

## Technical Validation

### At the start of data collection

We started data collection slowly and spent time discussing the first few papers assessed so that we were being consistent with our decisions as a group. The data category table^26^ and the standardised version of this table - the data dictionary (see the supplementary table 1)^26^ was a tool that helped us to maintain the same level of rigour throughout the assessment. The explanation column aimed at avoiding misinterpretation or overinterpretation of data categories by providing clear examples of the intended answers or particular aspects to consider when making decisions. Supplementary table 1 can also be found at: https://github.com/open-phytoliths/FAIR-assessment-data-paper-documentation/blob/main/FAIR-assessment-final-documents/Data-dictionary-FAIR-assessment-final.csv.

### Moderation during data collection

After each assessor had collected several papers worth of data, a moderation exercise was conducted. One of our team members looked independently at the three papers assessed by others and compared answers with the data collected by the other team members. We discussed the findings as a group and went back to recheck where any issues arose or changes needed to be implemented.

We also implemented a system of moderation for further data quality checks. After doing the data collection on each paper, if we were unsure about any of the data collected we made a comment in the readme file and highlighted the comments in yellow. This meant that the paper needed moderation by another team member.

A moderation meeting was conducted near the end of the data collection period to discuss the problematic papers and any data categories that had been hard to assess. Any changes agreed upon were then implemented on the whole dataset.

All discussions about the problems encountered during the evaluation can be found in this document in our Zenodo repository^26^. After manual data collection, we used the *roadoi* R package to capture the information for data categories 8 (open access) and 10 (What repository for green access article). We decided not to collect data for category 11 (Is it a signed up repo/ ResearchGate and Academia categories) using R as the *roadoi* package does not take into account academic social networking sites (ASNS) such as ResearchGate and Academia. Some information on academic social networks was entered in this data category manually but not for every article in our sample. These types of academic social networking sites are regarded as places to upload data and research articles by some researchers, but they do not comply with long-term repository requirements, among other responsible science requirements^34,35^. The results captured by using the *roadoi* R Package are presented in two files, one for each region, in their respective folders and the code is also available^26^.

### Data saturation

Once 100 papers had been assessed, data saturation was tested. A saturation method^36^ that assesses the thematic saturation of qualitative data was used to evaluate the two datasets separately (Europe and South America) in terms of representativity and saturation qualities.

The representativity was ensured by an adequate redundancy of the journal’s name.

The journal name, year (first published online), type of study, period/date and geographic location were the categories used to evaluate the data saturation.

The data saturation assessment showed we had collected enough data from the 100 papers to cease our data collection^26^.

## Usage Notes

The transparent recording of this dataset means that the methods used can be reproduced or replicated by others that want to conduct a FAIR assessment of the data in their own research community. Our data collection methodology could be used directly to look at phytolith data from different geographic areas of the world or easily adapted for use in related disciplines. There are other communities that have limited data sharing, especially in the use of open repositories^33,37^, and our methodology of assessing FAIR data would transfer well to these communities.

We also hope that our methodology can be reused by other related disciplines to undertake similar FAIR assessments for the improvement of data management and stewardship. Similar results for data sharing to those found for phytolith research by Karoune^23^ have also been found for another archaeobotanical discipline (macrobotanical remains)^37^, demonstrating that phytolith research is not alone in the need for change in this particular area.

We included articles from a wide range of phytolith study types and disciplines in this dataset, therefore data is not only related to archaeology and palaeoecological disciplines. This dataset can be reused by non-archaeologist phytolith researchers and help us to fill the gap between our approaches and methods.

This dataset can also be reused for teaching activities related to reproducibility and transparent recording as each step of our study is recorded and can be reused independently for different training courses about responsible science.

### Full description of the Zenodo repository files

#### FAIR assessment final documents

This folder contains the methodological files for our data collection, readme files, data collection forms and code used to collect data.

- Data-dictionary-FAIR-assessment-final.csv
- FAIR assessment data categories table
- Observations made during data assessment table
- Saturation and representativity of data collected.pdf
- Specifications table (Detailed overview of the study design).pdf
- README files
  - README file for European dataset
  - README file for South American dataset

- Data collection forms
  - FAIR Phytoliths Data Assessment Form – Europe
  - FAIR Phytoliths Data Assessment – South America
- *Roadoi* R package documentation
  - *roadoi* R package_Europe.csv
  - *roadoi* R package_South_America.csv
  - *roadoi* R package_Europe_code.md
  - *roadoi* R package_South_America_code.md

#### FAIR assessment trial

This folder contains information about the trial we conducted prior to starting our main data collection phase.

- Data collection methodology development document
- Data collection form for trial
- Table summarising how the FAIR principles were linked to a practical set of questions shaped specifically for phytolith research.pdf

data-raw

*roadoi* R package_South_America_code.md

- Contains 10.5281/zenodo.7851930/data raw
- Also contains the two separate raw datasets for Europe and South America as .csv files.

data-search

This folder contains the files of the results of the searches we conducted to produce lists of articles.

- contains .csv files of the searches conducted using publish or perish to get the list of relevant articles for Europe and South America articles.
  paper-tables
  This folder contains the tables in the article and as supplementary files.

**Table 1:** Specifications table (overview of the study design).

Subject	Phytolith research
**Specific subject areas**	Archaeology/Ecology/Geochemistry/Methodology (related to phytoliths)/Palaeoecology/Palaeontology/Plant physiology
**Dataset description**	This dataset contains information collected from 100 phytolith articles to inform guidelines for implementing the FAIR data principles in phytolith research - 50 selected from European studies and 50 selected from South American studies, over a 5 year publication time period (2016–2020).
**Data format**	Primary and secondary data
**Type of data**	Qualitative data
**How data were acquired**	Data were collected by six assessors in total (Emma Karoune, Carla Lancelotti, Juan José García-Granero, Javier Ruiz-Pérez, Marco Madella, Céline Kerfant). A trial of FAIR variables was conducted on five articles to check the criteria relevance by five assessors while the main data collection was carried out by four assessors.
	Access to the literature was carried out using *Publish or Perish* software (*Harzing, A.W. (2007) Publish or Perish, available*) to conduct two searches using the decided criteria (‘phytoliths’ and ‘Europe’ or ‘South America’) to produce a static list of papers. The first 100 articles selected from these lists using the inclusion criteria (‘primary data’, ‘phytoliths’ and ‘Europe’ or ‘South America’) were assessed on the basis of the availability and format of data and metadata presented. The R package *roadoi* (version 0.7.2) was used to transparently assess the accessibility of the articles. The languages of most of the studies are English and Spanish.
**Open science tools used**	*Publish or Perish* software (*Harzing, A.W. (2007) Publish or Perish, available*) - https://harzing.com/resources/publish-or-perish
	*roadoi* R package (version 0.7.2) - https://github.com/ropensci/roadoi
**Data availability**	Zenodo - 10.5281/zenodo.7215571
	GitHub repository - https://github.com/open-phytoliths/FAIR-assessment-data-paper-documentation
	Data is available under the Creative Commons Attribution 4.0 International licence
- Table-1-Specifications-table.pdf
- Supplementary-Table-1-Data-dictionary-FAIR-assessment.pdf

## Data Availability

All code and methods documentation for this study can be found at the Zenodo repository^26^. It can also be accessed through this link to our GitHub repository: https://github.com/open-phytoliths/FAIR-assessment-data-paper-documentation.
